# Investigation of Co-Assembly of Peanut Protein and Rice Protein: Effects on Protein Conformation and Immunoglobulin E Binding Capacity

**DOI:** 10.3390/foods14101699

**Published:** 2025-05-11

**Authors:** Qin Geng, David Julian McClements, Taotao Dai, Changhong Li, Zhihua Wu, Hongbing Chen

**Affiliations:** 1 State Key Laboratory of Food Science and Resources, Nanchang University, Nanchang 330047, China; 2 Sino-German Joint Research Institute, Nanchang University, Nanchang 330047, China; 3 Department of Food Science, University of Massachusetts, Amherst, MA 01003, USA

**Keywords:** peanut protein, rice protein, pH-driven treatment, allergenicity, structure

## Abstract

Reducing the allergenicity of food components through processing is attracting great attention in the food industry. This study investigated the structure and allergenicity of complexes formed from peanut protein (PP) and rice protein (RP) using the pH-driven method. The properties of the PP–RP complexes were investigated using morphological analysis, multi-spectroscopic characterization, IgE binding capacity analysis, and in vitro digestibility studies. Morphological analysis showed that the complexes lost their particulate structures after pH-driven treatment. Spectroscopic analysis showed that the pH-driven treatment promoted protein structural changes, making them more susceptible to degradation. There were also changes in the tertiary structures of the proteins in the complexes following the pH-driven treatment. The IgE binding capacity and digestibility studies suggested that the pH-driven treatment reduced the potential allergenicity of PP by altering its structure under gastrointestinal conditions. This study provides a promising approach for producing hypoallergenic peanut protein ingredients for use in the food industry.

## 1. Introduction

Food allergies are a global food safety and public health concern that have been increasing over time [1]. Peanut allergies are one of the most common types of food allergies [2]. According to the World Health Organization (WHO) and the Food and Agriculture Organization (FAO), peanuts and their products are one of the eight major food allergens [3]. Peanut allergies are characterized by immunoglobulin E (IgE)-mediated immune responses, which lead to gastrointestinal or systemic allergic reactions upon ingestion, which are mainly caused by allergens in the peanuts [4]. Peanut protein comprises 17 separate families of allergens [5]. The IgE epitopes present on these allergenic proteins are the fundamental antigen structures (epitopes) that the IgE antibodies recognize, which therefore play an important role in triggering peanut sensitization [6]. Variations in protein antigenicity can result from structural alterations, such as unfolding, aggregation, cross-linking, and chemical modifications [7]. Promoting substantial structural alterations of peanut proteins may therefore serve as a promising approach to decrease their allergenicity.

Extensive research has been conducted on using food processing techniques, such as heat, enzyme, or pressure treatments, to promote structural modifications of peanut proteins and thereby create more hypoallergenic peanut-based foods [8,9,10]. For instance, enzymatic cross-linking can alter the structure of proteins and reduce their allergenicity [11,12]. However, conventional processing operations have variable efficacy in reducing protein allergenicity and may also adversely impact the nutritional and sensory properties of foods [5]. Therefore, it would be advantageous to develop novel processing methods to reduce the allergenicity of peanut proteins without having adverse effects on their nutrition or functionality.

Recently, several researchers have focused on the conformational changes that occur in dietary proteins when the pH is shifted to neutrality after treatment in an extremely alkaline environment, a process known as pH-driven or pH-shifting treatment [13,14]. For instance, complexes have been formed from two different types of proteins by incubating them at pH 12 to induce protein unfolding and then adjusting them to neutral conditions to promote their refolding and assembly [15]. This process led to the formation of complexes with novel structures, whose properties could be controlled by varying the mass ratios of the two proteins, while both proteins retained their intact primary structures. Furthermore, our previous study demonstrated that modifying peanut proteins with other components, such as polyphenols, resulted in the formation of complexes between peanut proteins and ligands that significantly alter their allergenic potential [16]. Combining a non-allergenic food protein with an allergenic food protein may therefore be a novel approach for reducing peanut protein allergenicity. However, there is currently a lack of research on the application of the protein–protein co-assembly technique and the impact on peanut protein structure and allergenicity.

Rice proteins are crucial components in food products due to their exceptional nutritional value, functionality, potential health benefits, and potential application in plant-based foods. Moreover, they possess low allergenicity and are increasingly being utilized as nutritional and functional ingredients in foods developed for infants and the elderly [17]. Thus, we hypothesize that co-assembly of peanut protein and rice protein via the pH-shift method would reduce the potential allergenicity of peanut protein.

In this study, the co-assembly properties of peanut protein (PP) and rice protein (RP) through pH-shifting were therefore examined. The structural interplay between PP and RP, as well as the impact of RP on the allergenicity of PP, were investigated. The structural alterations of the PP before and after co-assembly with RP were investigated by using sodium dodecyl sulfate–polyacrylamide gel electrophoresis (SDS-PAGE), fluorescence spectroscopy, Fourier transform infrared spectroscopy (FT-IR), and ultraviolet-visible (UV-visible) spectroscopy. The impact of complexation on the allergenicity of the PP was then evaluated using an indirect competitive ELISA assay. Furthermore, the allergenicity of the digested products was also investigated after in vitro digestion under simulated gastrointestinal conditions. This study showed that the co-assembly of PP and RP could alter the structure and allergenicity of peanut protein. This information may be useful for developing peanut-protein-based food products with decreased allergenicity.

## 2. Materials and Methods

### 2.1. Materials

Peanut protein (PP) was obtained according to the method described in our previous study [16]. Rice protein (RP) (purity = 87.9%) was provided by Jiangxi Heng Ding Food Co., Ltd. (Jiangxi, China). 1-Anilinonaphthalene-8-sulfonic acid (ANS) and 4-nitrophenyl-N-acetyl-β-D-glucopyranoside were obtained from the Sigma-Aldrich Chemical Company (St Louis, MO, USA). Sera was purchased from Chongqing Mannualike Technology Co., Ltd. (Chongqing, China); detailed information about the sera is described in Table S1. HRP-conjugated goat anti-human IgE antibody was purchased from Bio-Rad Laboratories, Inc. (Hercules, CA, USA). Phosphate buffer (PB) was obtained from Solarbio Company (Beijing, China). The 96-well plate (31,121, LABSELECT) was obtained from Cyber Instruments Co., Ltd. (Beijing, China).

### 2.2. Preparation of PP–RP Complexes

The PP–RP complexes were prepared using the pH-driven approach according to the method described in a previous study [15]. Briefly, different concentrations of RP aqueous solution were added to a PP aqueous solution (10 mg/mL) to obtain mixtures with different PP–RP mass ratios (*w*/*w*) of 1:0.5, 1:1, and 1:2. The mixtures were then adjusted to pH 12.0 using 1 M of NaOH and magnetically stirred for 1 h to promote unfolding and dissolution of the proteins. Subsequently, the mixtures were adjusted to pH 7.0 using 0.1 M of HCl. The salt was then removed using an ultrafiltration tube with a molecular cut-off of 3500 Da. The supernatants were collected (which contained the PP–RP complexes), and then their properties were characterized using a variety of analytical methods. Pure PP and pure RP solutions were also prepared and treated under the same pH-shift conditions used to prepare the PP–RP composites. These samples were named pH-driven PP and RP, respectively. Native peanut protein (NPP) that had not undergone the pH-driven treatment was used as a control.

### 2.3. The Characterization of SDS-PAGE

SDS-PAGE analysis of NPP, PP, RP, and PP–RP complexes was performed according to a previously described method at a protein concentration of 1 mg/mL [18]. After electrophoresis, the gel was stained with Coomassie Brilliant Blue for 1 h, and the destained gel was visualized and photographed using a densitometer (GS-800, DNR Bio-Imaging Systems Ltd., Jerusalem, Israel).

### 2.4. Atomic Force Microscopy Analysis

The microstructure of the NPP, PP, RP, and PP–RP complexes was observed using atomic force microscopy (AFM) using a nano-probe cantilever tip (Bruker Nanoprobe, Camarillo, CA, USA) with a Multimode VIII microscope (Bruker Corporation, Billerica, MA, USA) [19]. Aqueous PP–RP composites were diluted with distilled water to 10 μg/mL. Then, approximately 5 μL samples were added dropwise onto a freshly cleaved mica sheet and dried at room temperature for 24 h prior to analysis.

### 2.5. Particle Size and Zeta Potential Analyses

The particle size and zeta potential measurements of the NPP, PP, RP, and PP–RP complexes were conducted using a Zetasizer Nano instrument (Malvern Instruments Ltd., Malvern, UK) according to a method described in a previous study [20]. All of the protein samples were diluted with distilled water to 0.1 mg/mL. The refractive indices of the protein and water used in the calculations were 1.45 and 1.33, respectively.

### 2.6. Surface Hydrophobicity Analysis

The surface hydrophobicity of the NPP, PP, RP, and PP–RP complexes was characterized based on the ANS probe fluorescence method using a fluorescence spectrophotometer (F-7000, Hitachi, Tokyo, Japan) with minor modifications [21]. Briefly, 3 mL of the sample was mixed with 30 μL of 8 mmol/L ANS solution. The resulting mixtures were then stored in the dark for 15 min. The samples were then poured into a quartz cuvette with a light path of 1 cm. The fluorescence spectra were acquired using an excitation wavelength of 390 nm and an emission wavelength range of 400 to 600 nm. The predicted theoretical value (TV) of the spectra of the mixed system was obtained by measuring the spectra of PP and RP solutions with the same PP or RP concentration as the PP–RP complexes.

### 2.7. Fluorescence Spectroscopy Analysis

The fluorescence spectra of the NPP, PP, RP, and PP–RP composites were measured using a fluorescence spectrophotometer (F-7000, Hitachi, Tokyo, Japan). The excitation wavelength was set at 295 nm, the slit width was set at 2.5 nm, and the emission spectrum ranged from 300 to 500 nm [5].

### 2.8. Ultraviolet-Visible (UV) Spectroscopy Analysis

The ultraviolet spectra of the NPP, PP, RP, and PP–RP composites were measured using a spectrophotometer (UV-2450, Shimadzu, Kyoto, Japan) from 200 to 400 nm using a 1.0 cm path length cell, following the method outlined by Chen and Zhang [20]. The absorption spectrum for a phosphate buffer blank was subtracted from that of the sample. Again, the predicted theoretical value (TV) of the spectra of the mixed system was obtained by measuring the spectra of PP and RP solutions with the same PP or RP concentration as the PP–RP complexes.

### 2.9. Fourier-Transform Infrared Spectroscopy (FTIR) Analysis

Approximately 1–2 mg of freeze-dried NPP, PP, RP, and PP–RP complexes was mixed with 140 mg of KBr and then ground into a powder. Then, the mixture was pressurized using a tablet press to form a transparent disk. A Fourier transform infrared spectroscopy instrument (Thermo Nicolet-5700, Waltham, MA, USA) was then used to measure the spectra over a wavenumber range from 500 to 4000 cm^−1^. PeakFit 4.12 (SeaSolve Software, Inc., Redlands, CA, USA) was used to deconvolute the amino I band (1700–1600 cm^−1^), and Gaussian fitting was performed on the deconvolution spectra using the iterative method. To be specific, β-sheets were in 1600–1640 cm^−1^, random coil was in 1640–1650 cm^−1^, α-helixes were in 1650–1660 cm^−1^ and β-turns were in 1660–1700 cm^−1^. The secondary structure content of the proteins was calculated using the area ratio method described by Cao, Liao, and Zhang [22].

### 2.10. Differential Scanning Calorimetry (DSC) Analysis

The thermal behavior of NPP, PP, RP, and PP–RP complexes under a nitrogen atmosphere was evaluated using a differential scanning calorimeter (DSC 822E, Mettler Toledo, Zurich, Switzerland) according to a method described previously [23]. Briefly, powdered protein samples (~7 mg) were weighed and hermetically sealed in aluminum pans and then heated at a rate of 10 °C/min from 25 to 160 °C. The peak temperature (T_d_) and enthalpy change (Δ*H*) of any thermal transitions in the protein samples were then calculated using the instrument’s software.

### 2.11. Enzyme-Linked Immunosorbent Assay (ELISA) Analysis

The IgE binding activity of the PP–RP composites was assessed using an indirect competitive enzyme-linked immunosorbent assay (ELISA) following a previously described method [24]. Briefly, the PP was diluted using coating buffer (0.05 M of carbonate–bicarbonate buffer, pH 9.6), the diluted PP sample (100 μL) was added to individual wells of a 96-well plate and incubated overnight at 4 °C. After removing the sample solution, the wells were washed five times with PBS Tween-20 (PBST) for 5 min each time. Subsequently, blocking buffer (5% *w*/*v* skimmed milk in PBS, 250 μL/well) was added and incubated at 37 °C for 2 h to block nonspecific binding sites. Following removal of the blocking buffer, PBST washes were performed five times for 5 min each to remove any unbound components. Next, a mixture of 50 μL of the PP–RP composite sample (concentration range: 0.5–20 μg/mL) and 50 μL of serum dissolved in PBS from peanut-allergic patients (diluted at a ratio of 1:300) was added to each well (100 μL), followed by incubation at 37 °C for 1 h to allow for specific binding between IgE present in the patient sera and the allergen. The wells were then washed again with PBST five times for 5 min each before adding HRP-conjugated goat anti-human IgE antibody dissolved in PBS (100 μL, diluted at a ratio of 1:20,000). This step involved incubating the plates at 37 °C for one hour to enable binding between HRP-conjugated secondary antibody and captured IgE antibodies on solid phase surfaces within each well. After washing the wells as described previously, TMB developer solution (100 μL per well) was added and allowed to react with bound HRP conjugates under incubation at 37 °C for 15 min. Finally, stopping solution (50 μL, H_2_SO_4_, 2 M) was added to stop the enzyme-catalyzed reaction. The absorbance at 450 nm was measured using a UV-visible spectrophotometer (UV-2450, Shimadzu, Kyoto, Japan). It should also be noted that the concentration of protein here and elsewhere in the entire text was determined through the classic Coomassie Brilliant Blue method.

### 2.12. Digestibility Analysis

The digestibility analysis was performed based on the in vitro gastrointestinal tract model described by Zhang, Wu, and Li [18].

Initially, simulated gastric fluid (SGF) solution (1 mL) was prepared, which contained 170 mM of NaCl (Damao Chemical Reagent Factory, Tianjin, China) and 25,000 U of pepsin (Sigma, Saint Louis, MO, USA) adjusted to pH 1.2. This solution was then placed in test tubes and warmed to 37 °C for 5 min. Then, 1 mL of PP–RP complex solution (1 mg/mL) was mixed with 0.4 mL of SGF solution. The resulting mixture was then incubated for 80 min at 37 °C using a ratio of pepsin activity to substrate protein amount of 10 U: 1 μg. Gastric digestion was terminated by adjusting the pH to 7.5 by adding 0.2 M of Na_2_CO_3_. After intervals of 0, 10, 20, 40, and 80 min, 20 μL samples were collected from the gastric fluids for Tricine-SDS-PAGE analysis.

The simulated intestinal fluid (SIF) solution was prepared according to the US Pharmacopoeia. The SIF solution was then mixed with the SGF solutions collected after 80 min of gastric digestion using the following volume ratio: SIF:SGF = 3:1 (*v*/*v*). After being incubated for 40 min at 37 °C, digestion was stopped by heating the samples at 100 °C for 5 min. During intestinal digestion, 20 μL samples were collected at intervals of 0, 5, 10, 20, and 40 min for Tricine-SDS-PAGE analysis.

### 2.13. Statistical Analysis

The results were expressed as mean values ± standard deviations. Statistical analysis was performed using IBM SPSS (version 26.0, IBM Inc., Armonk, NY, USA). The Duncan test was utilized to analyze significant differences between means (*p* < 0.05) using a one-way ANOVA test.

## 3. Results and Discussion

### 3.1. Characterization of the Co-Assembled Structures

**Figure 1:**
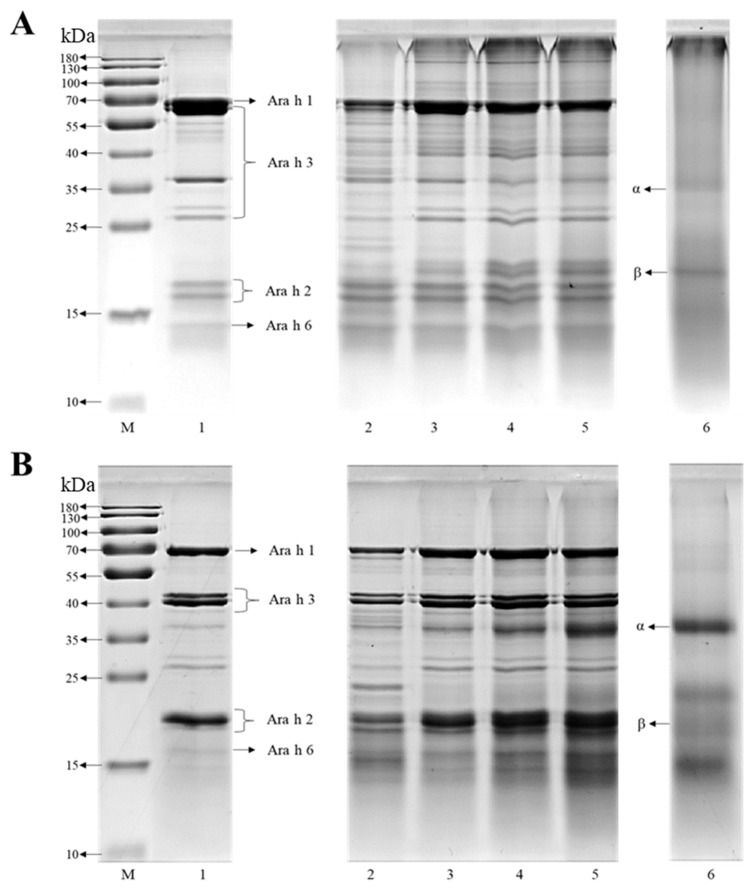
SDS-PAGE of native peanut protein (NPP), peanut protein (PP), rice protein (RP), and their composites with different mass ratios of PP: RP. (**A**) Denaturing non-reducing SDS-PAGE. (**B**) Reduced SDS-PAGE. Lane M is the marker, lanes 1, 2, and 6 are NPP, PP, and RP, and lanes 3–5 are composites prepared at PP: RP = 1:0.5, 1:1, and 1:2, respectively.

Denaturing non-reducing (Figure 1A) and reducing (Figure 1B) SDS-PAGE analyses were used to characterize the subunits in the proteins based on their molecular weights. Bands corresponding to NPP, PP, and RP are shown in lanes 1, 2 and 6, respectively. Under non-reducing conditions (without β-mercaptoethanol), several characteristic bands associated with major peanut allergens were observed in the SDS-PAGE profiles of NPP (Figure 1A), including Ara h 1 (64 kDa), Ara h 3 (43/38/36/24 kDa), Ara h 2 (17–20 kDa), and Ara h 6 (15 kDa) [25]. Compared to the NPP sample, the pH-shifted PP sample (lane 2) showed accumulation of extremely large proteins with a major band appearing at the top of the separation gel, which might be attributed to disulfide cross-linking of the protein molecules under strongly alkaline conditions [26]. Furthermore, the intensity of the band corresponding to Ara h 1 and Ara h 3, with molecular mass over 55 kDa, decreased, while some new bands appeared around 20 and 40 kDa after the pH-shift treatment, indicating both the formation of high MW protein polymers and the generation of peptide fragments. Conversely, no obvious changes were observed in the protein subunits of Ara h 2 and Ara h 6.

Under reducing conditions (with β-mercaptoethanol), the large protein polymers and aggregates appeared to have significantly dissociated, as suggested by the lower intensity bands observed at higher MWs (Figure 1B). Compared to the native proteins, a visible increase in protein bands around 20 and 38 kDa was observed in the PP group, which suggested that protein hydrolysis or other alterations had occurred. These conformational changes may lead to favorable changes in the allergenicity of the peanut proteins. For RP, two prominent bands at 17 and 30 kDa were observed, corresponding to the acid (α) and base (β) subunits of glutelin, respectively, which are the predominant subunits in rice proteins [15]. After complexation (lines 3–5) (Figure 1A), the MW band of the peanut protein observed around 20 kDa became fainter or disappeared, while the MW band around 60 kDa became more intense. It should be noted that as the RP ratio increases, there is a noticeable increase in the β band of RP near 20 kDa (lanes 3–5, Figure 1A) and the α band in Figure 1B. These changes can strongly support the formation of the complexes. In addition, more intense bands appeared with MWs exceeding 130 kDa after complexation compared to the PP (line 2) under non-reducing SDS-PAGE conditions. These results suggest that polymerization occurred between PP and RP. However, under reducing conditions, all of the characteristic bands of PP and RP remained intact in the composites. This result is consistent with that reported by other researchers [27], who also reported that both proteins remained intact in composites and that their molecular integrity was retained.

### 3.2. Morphological Analysis

**Figure 2:**
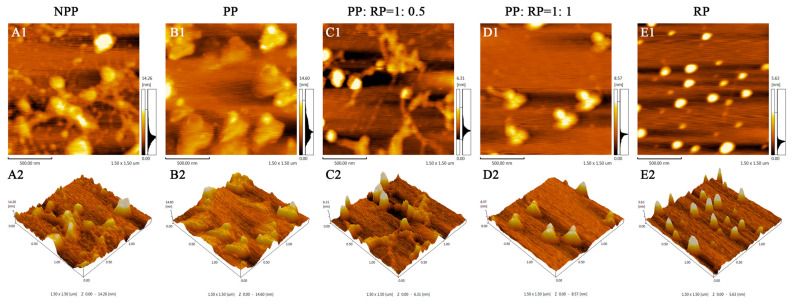
The AFM morphologies of native peanut protein (NPP), peanut protein (PP), rice protein (RP), and their composites. (**A1**,**A2**)–(**E1**,**E2**) are NPP, PP, PP:RP = 1:0.5, PP:RP = 1:1, and RP, respectively.

The microstructures of the NPP, PP, RP, and composite samples were recorded using atomic force microscopy (Figure 2). The AFM images show that all of the samples contained protein aggregates that were a few hundred nanometers in diameter, but the microstructures of these aggregates depended on protein composition and pH shifting. The native peanut protein (NPP) sample contained a mixture of large spheroidal protein aggregates with some irregular filamentous structures between them (Figure 2A1,A2). The pH-shift-treated peanut protein (PP) sample contained larger protein aggregates that had more uniform dimensions and shapes (Figure 2B1,B2). This result suggests that exposing PPs to alkaline conditions and then bringing them back to neutral pH conditions changed their spatial organization. It is likely that the strong electrostatic repulsion between the highly negatively charged protein molecules at pH 12 promoted the dissociation of any aggregates [28]. Then, the protein molecules assembled into more uniform aggregates when the pH was neutralized. The pH-shift-treated rice protein (RP) samples also contained uniform aggregates, but these were considerably smaller than those in the PP samples (Figure 2E1,E2), which may be due to differences in the surface chemistries and molecular interactions of the two different sources of proteins. The formation of uniform protein aggregates in RP dispersions after a pH-shift treatment has also been reported in previous studies [29].

The microstructures of samples containing mixtures of PP and RP were also examined. At a PP:RP ratio of 1: 0.5 *w*/*w*, the samples contained some spheroidal particles, as well as numerous irregular fibrous structures (Figure 2C1,C2). In contrast, at a PP:RP ratio of 1:1 *w*/*w*, the samples contained particles with more uniform shapes (“trimer” structures) and dimensions (around 200–300 nm) (Figure 2D1,D2). These results suggest that complexes were formed that had morphologies that depended on the protein ratio present. Wang, Li, and Feng [15] reported that increasing the proportion of pea protein to rice protein in binary mixtures led to the formation of more uniform complexes, which is consistent with our results.

### 3.3. Particle Size and Zeta Potential

**Figure 3:**
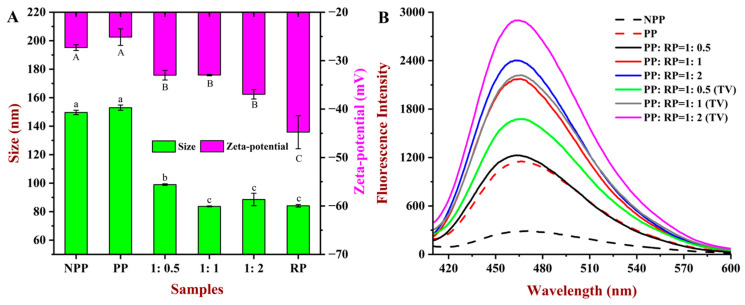
The particle size and zeta potential values of peanut protein (PP), rice protein (RP), and their composites (**A**). “A”, “B”, “C”, and “a–c” indicated the values of zeta potential and size had a significant difference (*p* < 0.05) between different samples. Changes in ANS fluorescence intensity of PP, RP, and their composites (**B**). The theoretical value (TV) of absorbance was the direct addition of the experimental values of the proteins with corresponding concentrations.

The particle size and zeta potential values of the PP, RP, and composite samples are shown in Figure 3A. The NPP sample exhibited a relatively large average particle diameter (150 nm) and negative zeta potential (−27.3 mV). After the pH-shift treatment, the particle size of the PP sample remained relatively high (153 nm), which can be attributed to the formation of large uniform aggregates, as seen in the AFM images (Figure 2). In addition, there was a slight decrease in the magnitude of the negative charge on the protein aggregates in the PP sample after the pH-shift treatment (−25.0 mV), which suggested that the strongly alkaline conditions changed the surface chemistry of the aggregates. The protein aggregates in the RP samples had a much smaller average particle diameter (84 nm) and higher negative charge (−45 mV) than those in the PP samples. The smaller particle size of the RP samples may have been because the higher negative charge on the individual rice proteins generated a stronger electrostatic repulsion between the protein molecules, which limited the size of the protein aggregates formed under neutral conditions.

The particle size and zeta potential of PP–RP complexes with different protein compositions were also measured (Figure 3A). The average particle diameter of all of the complexes was relatively small (85–100 nm), and their zeta potential values were relatively large (−33–37 mV). With a change in the PP:RP ratio from 1:0.5 to 1:1 *w*/*w*, there was a slight increase in the magnitude of the negative charge on the complexes, which may have been due to the stronger negative charge on the rice proteins. However, when the PP:RP ratio was further increased to 1:2 *w*/*w*, there was no further change in the particle charge, which may have been because the additional rice proteins were not incorporated into the complexes or because the surface composition of the complexes did not change. The size of the PP:RP complexes also depended on their composition, being 99.0, 83.7, and 88.6 nm for the PP:RP = 1:0.5, 1:1, and 1:2 samples, respectively. There was no significant difference in the average diameter of the composites with the 1:1 and 1:2 PP:RP ratios, which again suggests that the additional rice proteins may not have been incorporated into the complexes.

Overall, these results show that complexation of PP with RP reduced the average size and increased the zeta potential of the particles formed after the pH-shift treatment, which should improve their water dispersibility and storage stability by inhibiting particle aggregation and sedimentation. Notably, the particle size and zeta potential of the composites did not change when the PP–RP ratio increased from 1:1 to 1:2, indicating that complexes must have already being formed at the lower ratio [15].

### 3.4. Surface Hydrophobicity

Information about the interfacial properties of the proteins was obtained by measuring their surface hydrophobicity (*H*_0_) using an ANS probe (Figure 3B), which is a surface-active fluorescent dye that has affinity for hydrophobic patches on the surfaces of protein molecules dissolved or dispersed in water [21]. The maximum fluorescence intensity increased after the native protein (NPP) was subjected to the pH-driven treatment (PP), which indicates that the polarity of the microenvironment surrounding the tryptophan and tyrosine residues increased. This result suggests that there were changes in the conformation and/or aggregation state of the proteins after the alkaline treatment. Figure 3B shows that as the RP concentration in the complexes was increased, the surface hydrophobicity of the complexes increased. However, this increase was significantly lower than the theoretical value (TV), which was the sum of the individual protein values at the corresponding concentrations. This discrepancy might be attributed to the interactions between the two proteins when the complexes are formed. Some hydrophobic groups were concealed within the complexes. This revealed details about the hydrophobic reactive side chains that link PP and RP together. Taken together, the morphological and surface hydrophobicity measurements suggested that the PP and RP formed complexes [29].

### 3.5. Spectroscopy Analysis

#### 3.5.1. Fluorescence Spectroscopy

**Figure 4:**
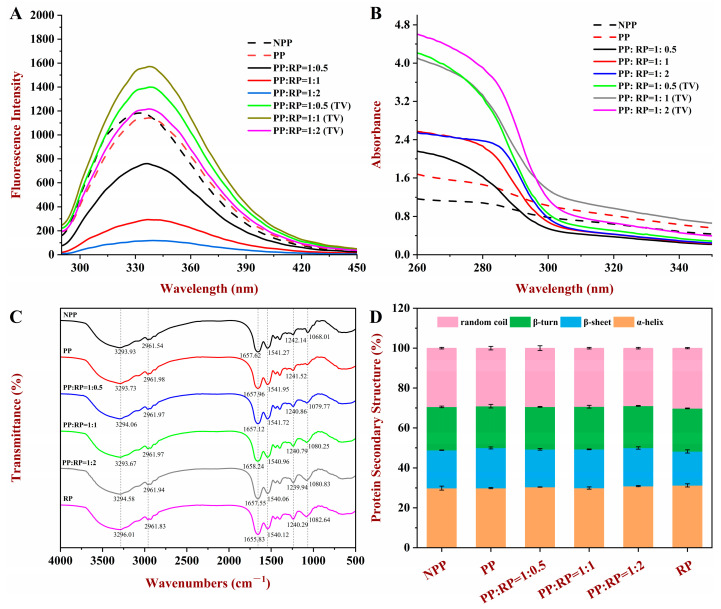
Endogenous fluorescence intensity (**A**), UV spectroscopy (**B**), FT-IR spectrum (**C**), and secondary structure (**D**) of native peanut protein (NPP), peanut protein (PP), rice protein (RP), and their composites.

The fluorescence spectra of the various proteins were measured (Figure 4A). The maximum fluorescence intensity of the native peanut proteins (NPPs) was significantly higher than that of the peanut proteins that had been subjected to the pH-driven treatment (PP), indicating that the tertiary structure of the proteins had been changed after they were subjected to the alkaline treatment. The fluorescence intensity of the PP was significantly quenched after the addition of the RP (Figure 4A), indicating that the two types of proteins interacted with each other [27]. The change in the fluorescence spectra after complex formation may have been due to alterations in the conformation of the proteins or due to their aggregation, which altered the exposure of the tryptophan and tyrosine groups to water.

#### 3.5.2. UV Spectroscopy

UV spectroscopy is also sensitive to changes in the molecular conformation of proteins. Consequently, it was used to provide additional insights into the interactions between the PP and the RP. The UV spectra of the PP and RP samples had a peak around 285 nm (Figure 4B), which can mainly be attributed to absorption of ultraviolet light by tryptophan and tyrosine residues [16]. The theoretical value (TV) of absorbance was calculated as the sum of the experimental values of the absorbances for individual protein solutions with similar concentrations. The addition of RP increased the UV maxima (U_max_) of PP, although it remained lower than the TV, suggesting interactions between the PP and the RP. When the amount of RP was increased, the absorbance of the PP–RP complexes showed no significant change. Overall, these results again suggest that the two proteins formed complexes with each other [30].

#### 3.5.3. FTIR Spectroscopy

FTIR spectroscopy was used to determine the secondary structures of the proteins (Figure 4C,D). All of the samples had similar FTIR profiles, indicating that there were no significant changes in the backbone structure of the polypeptides. The PP, RP, and complex samples displayed three significant peaks in the wavenumber range from 1700 to 1200 cm^−1^, which corresponded to Amide-I (1657 cm^−1^), Amide-II (1541 cm^−1^), and Amide-III (1241 cm^−1^) [22]. The secondary structure content of the proteins was calculated by analyzing the spectra from 1700 to 1600 cm^−1^ (Amide-I band) using PeakFit software (AISN Software Inc., Mapleton, OR, USA). This part of the spectra is sensitive to the amounts of α-helix, β-sheet, β-turns, and random coil regions within a protein [31]. As shown in Figure 4D, the protein samples did not show any shift in amide-I peak after treatment, indicating that the secondary structures of the proteins were not significantly affected by pH-driven treatment or RP addition. A similar phenomenon was reported for soy proteins after they were subjected to strong alkaline conditions [32]. The findings may suggest that the interactions between the secondary structures of two proteins were what caused the composites.

### 3.6. DSC Analysis

**Figure 5:**
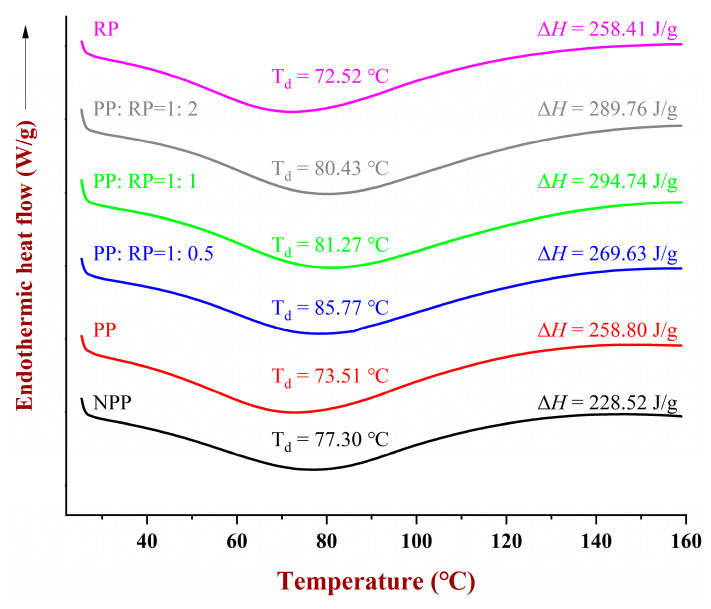
DSC analysis of native peanut protein (NPP), peanut protein (PP), rice protein (RP), and their composites.

Differential scanning calorimetry was used to study the thermal stability of the proteins. The peak temperature (T_d_) and enthalpy change (Δ*H*) associated with thermal denaturation provide information about protein stability and the degree of denaturation. As presented in Figure 5, the NPP had a single endothermic peak at 77.3 °C. A lower denaturation temperature (73.5 °C) and higher enthalpy change (258.8 J/g) were observed for the pH-shift-treated samples (PP), which can be attributed to the disruption of protein bonds and the exposure of hydrophobic groups under strongly alkaline conditions [33]. The denaturation temperature of the pH-shift-treated rice proteins (72.5 °C) was only slightly lower than that of the peanut proteins (73.5 °C), suggesting that they had slightly lower thermal stability.

Interestingly, the denaturation temperature of the complexes (80.4 to 85.8 °C) was much greater than that of the individual pH-shift-treated proteins (72.5–73.5 °C), which suggests that complexation increased the thermal stability of the proteins. The thermal denaturation temperature increased as the fraction of RP in the complexes increased, which again suggests that the presence of the rice proteins improved the thermal stability of the peanut proteins. This effect may have been due to the formation of hydrophobic bonds between the two different types of protein molecules in the complexes. The Δ*H* values provide an indication of the thermal energy required to cause the unfolding of the proteins. Compared to the native PP, the treated proteins had higher enthalpy values, again indicating that the pH-driven treatment and complex formation improved the thermal stability of the peanut proteins.

### 3.7. In Vitro Allergenicity Assessment

**Figure 6:**
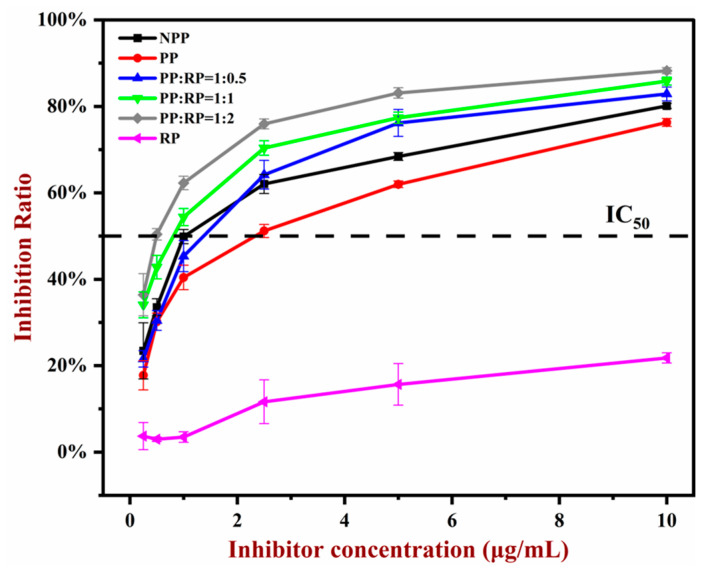
The IgE binding capacity of native peanut protein (NPP), peanut protein (PP), rice protein (RP), and their composites.

The effects of the pH-driven treatment and complexation with rice proteins on the IgE binding ability of the peanut protein was examined using indirect competitive ELISA (Figure 6). In this assay, the IC_50_ value, which is the protein concentration required to inhibit the binding by 50%, characterizes the strength of the IgE binding ability to the allergen. A lower IC_50_ value indicates a stronger IgE binding ability of the allergen. Compared to native PP, the IC_50_ value of the pH-shift-treated PP increased significantly, showing that the IgE binding capacity of the PP was reduced due to being exposed to strong alkaline conditions. This result suggests that the pH-driven treatment has the potential to alter the structure or conformation of the IgE epitopes of the peanut proteins, thereby reducing their potential allergenicity.

The IC_50_ values of the PP–RP complexes were significantly lower than those of the PP alone, with the IC_50_ value decreasing with increasing RP content. This result indicates that the IgE binding capacity of the PP increased after complexation with the RP. This effect may have been because conformational epitopes remained after the pH-shift treatment and became more exposed to the surrounding aqueous phase, thereby leading to an increase in IgE binding.

### 3.8. Digestibility

**Figure 7:**
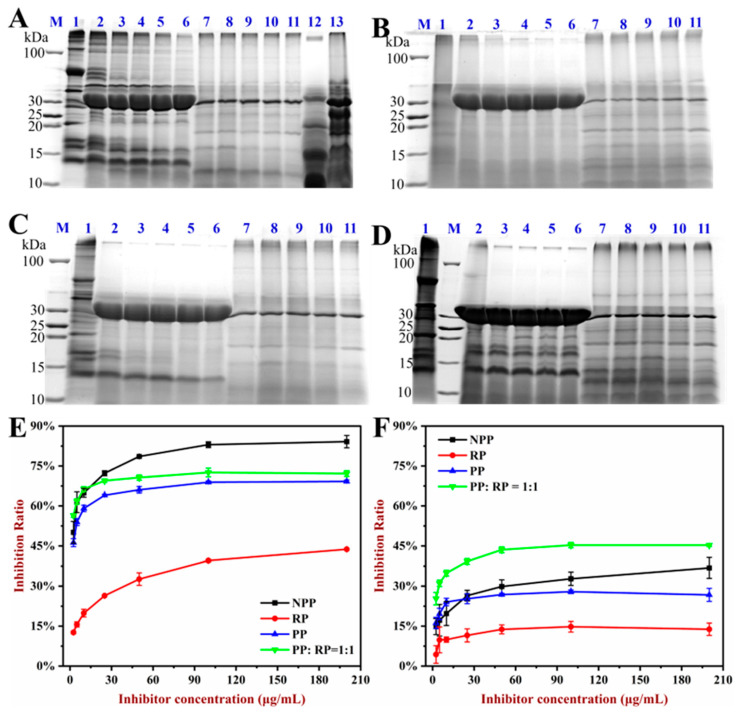
The digestibility results of native peanut protein (NPP), peanut protein (PP), rice protein (RP), and PP:RP = 1:1. (**A**–**D**) are the SDS-PAGE of digested NPP, RP, PP, and PP:RP = 1:1, respectively. Lane M, protein marker; lane 1, undigested protein; lanes 2–6, simulated gastric digestion for 0, 10, 20, 40, and 80 min, respectively; lanes 7–11, simulated intestinal digestion for 0, 5, 10, 20, and 40 min, respectively; lanes 12–13, simulated gastric digestion solution and simulated intestinal digestion solution, respectively. (**E**) The IgE binding capacity of SGF-digestive NPP, RP, PP, and PP:RP = 1:1. (**F**) The IgE binding capacity of SIF-digestive NPP, RP, PP, and PP:RP = 1:1.

The allergenicity of proteins is also impacted by their digestibility within the gastrointestinal tract [34]. The digestibility results of the NPP, RP, PP, and PP:RP (1:1) samples are shown in Figure 7. The 1:1 PP:RP sample was chosen for these studies as a representative of the complexes. Figure 7A–D show that some band intensities of the proteins decreased significantly or disappeared with increasing digestion time. Compared to NPP, some of the smaller molecular weight bands in the PP (corresponding to Ara h 2 and Ara h 6) disappeared faster (Figure 7A,C). This finding suggests that the pH-driven-treated peanut protein was more easily digested than the native version. Notably, some of the bands in the PP:RP sample that were associated with smaller molecular weight proteins did not significantly change during SGF digestion, indicating that these polypeptides had strong resistance to pepsin digestion.

The effects on the IgE binding capacity of the proteins after SGF and SIF digestion are shown in Figure 7E,F. After digestion, the gastrointestinal digestion products of the PP showed significantly decreased IgE binding capacities compared to those of the NPP. This effect could have been because the pH-driven treatment caused some unfolding of the peanut proteins, thereby increasing their susceptibility to digestion into short peptides, destroying the IgE binding epitopes, and decreasing sensitization to allergens [18]. However, the PP–RP complexes exhibited lower digestibility and an increase in IgE binding capacity after gastrointestinal digestion. These findings suggest that complex formation reduced the ability of the proteases to access the peptide bonds, thereby limiting protein digestion, which may have important effects on the allergenicity of the proteins.

## 4. Conclusions

In this work, the ability of peanut protein (PP) and rice protein (RP) to form complexes during a pH-shift treatment was examined, and the effect of complexation on the potential allergenicity of the peanut proteins was elucidated. The alkaline conditions used, as well as the complexation with rice protein, changed the tertiary structure of the peanut proteins. The IgE binding capacity of the peanut proteins was reduced after the pH-driven treatment. In contrast, the binding capacity increased after the peanut proteins formed complexes with the rice proteins. Peanut protein had a higher in vitro digestibility and lower IgE binding capacity after pH-driven treatment. These effects were attributed to alterations in the structure and aggregation of the proteins when they were exposed to strongly alkaline conditions and then neutralized, which changed the accessibility of the proteins to proteases in the gastrointestinal fluids. Therefore, the pH-driven treatment appears be a promising approach for improving the hypoallergenicity of peanut proteins. In contrast, this study showed that the formation of complexes between peanut protein and rice protein increased the allergenicity of the peanut proteins, which may have been because complexation increased the resistance of the peanut proteins to digestion. In other words, pH treatment alone shows promise for hypoallergenic applications, whereas indiscriminate complexation may inadvertently restore allergenicity. The knowledge obtained in this study may be useful for the development of more hypoallergenic peanut-protein-based foods in the future.

## Data Availability

The original contributions presented in this study are included in the article/Supplementary Materials. Further inquiries can be directed to the corresponding author.

## Supplementary Materials

The following supporting information can be downloaded at https://www.mdpi.com/article/10.3390/foods14101699/s1, Tabel S1: Information of 11 peanut allergy patients.
