# Supplementary material for: Investigation of Co-Assembly of Peanut Protein and Rice Protein: Effects on Protein Conformation and Immunoglobulin E Binding Capacity
Source: Foods. 2025 May 11;14(10):1699. doi: 10.3390/foods14101699 (PMC12111746; doi:10.3390/foods14101699)
Supplement: Supplementary file 1 [file foods-14-01699-s001.zip › foods-3617189-supplementary.pdf]

foods

Article

Investigation of co-assembly of peanut protein and rice protein: Effects on protein conformation and IgE-binding capacity

Qin Geng [1], David Julian McClements [3], Taotao Dai [1], Changhong Li [1], Zhihua Wu [1,2,] *, Hongbing Chen [1,2]

[1] State Key Laboratory of Food Science and Resources, Nanchang University, Nanchang, China;
[2] College of Food Science and Technology, Nanchang University, Nanchang, China;
[3] Sino-German Joint Research Institute, Nanchang University, Nanchang, China;
* Correspondence: wuzhihua@ncu.edu.cn;

Tabel S1: Information of 11 peanut allergy patients.

Number	Gender	Age	Clinical symptoms	Total IgE level (IU/mL)	Specific IgE level for peanut (IU/mL)
1	Male	21	Elevated IgE	238	20.6
2	Male	23	Allergic rhinitis	ND	>=100
3	Male	19	Allergic rhinitis	95.605	42.299
4	Female	21	Allergic rhinitis	233	55.6
5	Male	23	Allergic rhinitis, multiple allergies	9082.5	46.3
6	Female	49	Allergic rhinitis, asthma, celiac disease, multiple allergies	370	77.4
7	Male	15	Allergic rhinitis, multiple allergies	ND	24.3
8	Male	21	Allergic rhinitis, food allergy	977.378	>=100
9	Male	26	Allergic rhinitis	ND	>=100
10	Female	23	Allergic rhinitis, multiple allergies	852	43.3
11	Male	22	Allergic rhinitis, asthma, multiple allergies	3532	47.9
